# Enteropathogenic *Escherichia coli* (EPEC) inactivate innate immune responses prior to compromising epithelial barrier function

**DOI:** 10.1111/j.1462-5822.2007.00923.x

**Published:** 2007-08-01

**Authors:** Marie-Hélène Ruchaud-Sparagano, Marc Maresca, Brendan Kenny

**Affiliations:** Institute for Cell and Molecular Biosciences, Medical School, University of Newcastle Framlington Place, Newcastle upon Tyne, NE2 4HH, UK.

## Abstract

Enteropathogenic *Escherichia coli* (EPEC) infection of the human small intestine induces severe watery diarrhoea linked to a rather weak inflammatory response despite EPEC's *in vivo* capacity to disrupt epithelial barrier function. Here, we demonstrate that EPEC flagellin triggers the secretion of the pro-inflammatory cytokine, interleukin (IL)-8, from small (Caco-2) and large (T84) intestinal epithelia model systems. Interestingly, IL-8 secretion required basolateral infection of T84 cells implying that flagellin must penetrate the epithelial barrier. In contrast, apical infection of Caco-2 cells induced IL-8 secretion but less potently than basolateral infections. Importantly, infection of Caco-2, but not T84 cells rapidly inhibited IL-8 secretion by a mechanism dependent on the delivery of effectors through a translocation system encoded on the locus of enterocyte effacement (LEE). Moreover, EPEC prevents the phosphorylation-associated activation of multiple kinase pathways regulating IL-8 gene transcription by a mechanism apparently independent of LEE-encoded effectors and four non-LEE-encoded effectors. Crucially, our studies reveal that EPEC inhibits the capacity of the cells to secrete IL-8 in response to bacterial antigens and inflammatory cytokines prior to disrupting barrier function by a distinct mechanism. Thus, these findings also lend themselves to a plausible mechanism to explain the absence of a strong inflammatory response in EPEC-infected humans.

## Introduction

Enteropathogenic *Escherichia coli* (EPEC) infection of the small intestine is responsible for triggering severe watery diarrhoea in millions of people resulting in several hundred thousand infant deaths each year (Nataro and Kaper, 1998; Kaper *et al*., 2004; Chen and Frankel, 2005). Disease is dependent on EPEC interaction with enterocytes lining the small intestine triggering the localized loss (effacement) of absorptive microvilli and accumulation of host cytoskeletal proteins into pedestal-like structures beneath the adherent non-invasive bacteria. The ability of this pathogen to induce these responses is dependent on the ∼35 kb locus of enterocyte effacement (LEE) which encodes, among other things, a type three secretion system (TTSS), EPEC-secreted proteins (Esp), injected ‘effector’ proteins and the surface protein, Intimin (Chen and Frankel, 2005; Dean *et al*., 2005). The TTSS and EspA,B,D proteins function together to form a ‘molecular syringe’ that delivers effector proteins (both LEE- and non-LEE-encoded) into host cells to subvert signalling cascades (Chen and Frankel, 2005; Dean *et al*., 2005; Tobe *et al*., 2006). The only effector protein so far shown to be essential for the disease process is the LEE-encoded translocated intimin receptor (Tir) (Marches *et al*., 2000; Deng *et al*., 2003). Delivery of Tir results in its insertion into the host plasma membrane in a hairpin-like conformation where its extracellular domain acts as a receptor for the LEE-encoded outer membrane protein, Intimin, to mediate intimate adherence (Kenny *et al*., 1997; Kenny, 1999). The essential nature of the TTSS/Esp ‘molecular syringe’, Intimin and Tir molecules in disease (Marches *et al*., 2000; Deng *et al*., 2003) presumably reflects their roles in Tir delivery and Tir/Intimin-mediated intimate adherence. However, this interaction with Intimin also unleashes Tir signalling functions leading to pedestal formation, phosphorylation of a host phospholipase and downregulation of EPEC-induced actin rearrangements (Kenny, 2002; Dean *et al*., 2005). Another two LEE effectors, Map (mitochondrial-associated protein) and EspF target mitochondria where they interfere with organelle activity and/or shape (Kenny and Jepson, 2000; Nagai *et al*., 2005; Papatheodorou *et al*., 2006) but also possess functions independent of mitochondrial targeting (Dean *et al*., 2005). Relatively little is known about the LEE-encoded EspG, EspH and EspZ effectors except that EspG plays a role, along with its non-LEE homologue Orf3, in disrupting microtubules while EspH impacts on the dynamics of EPEC-induced cytoskeletal rearrangements (Matsuzawa *et al*., 2004; Dean *et al*., 2005; Kanack *et al*., 2005).

Enteropathogenic *E. coli* is the prototypic member of the attaching and effacing family of pathogens that carry the LEE pathogenicity island, including the human pathogen enterohaemorrhagic *E. coli* (EHEC) with others colonizing rabbits (REPEC), pigs (PEPEC), ruminants (EHEC) and mice (*Citrobacter rodentium*). Importantly, 177 homologous ‘pathogenicity islands’ have been identified in EHEC and EPEC genome sequences with the potential to encode thousands of virulence factors (Perna *et al*., 2001; Spears *et al*., 2006), with a recent study experimentally defining 39 EHEC secreted/translocated proteins (Tobe *et al*., 2006). Studies to date on non-LEE-encoded effectors have revealed properties such as (i) targeting the Golgi apparatus, (ii) interfering with cellular division and (iii) playing roles in bacterial colonization (Chen and Frankel, 2005; Dean *et al*., 2005).

An unexpected complexity in EPEC pathogenicity has recently been revealed by the finding that LEE and non-LEE effectors can function together, and in various combinations, to subvert host cellular processes (Matsuzawa *et al*., 2004; Dean *et al*., 2005). For example, the LEE-encoded Map, EspF, Tir and Intimin proteins act together in different combinations to trigger several disease-related changes including loss of absorptive microvilli (Dean *et al*., 2006), rapid inactivation of the major small intestinal water pump (Dean *et al*., 2006) and disruption of epithelial barrier function (Dean and Kenny, 2004). Importantly, the postulated roles for Map and EspF in disrupting barrier function have been validated *in vivo* (Shifflett *et al*., 2005; Ma *et al*., 2006). However, this ability to compromise barrier integrity raises the question of why EPEC human infection is linked to a rather weak inflammatory response compared with enteric pathogens such as *Shigella* (Ashkenazi *et al*., 1983; Miller *et al*., 1994; Nataro and Kaper, 1998) given the association of barrier dysfunction with inflammatory disease (Mullin *et al*., 2005). *In vitro* studies using non-differentiated host cells have identified EPEC flagellin as a potent pro-inflammatory mediator that induces the expression, and subsequent release, of the chemokine interleukin (IL)-8 that facilitates the recruitment of inflammatory-associated immune cells such as polymorphonuclear leucocytes (Savkovic *et al*., 1996; 1997; 2001; 2003; Czerucka *et al*., 2001; Zhou *et al*., 2003; Borthakur *et al*., 2006). Interestingly, while most of these studies conclude that EPEC triggers a pro-inflammatory IL-8 secretory response, one indicated that EPEC may inhibit IL-8 secretion (Hauf and Chakraborty, 2003). This possibility is supported by our findings that EPEC infection of differentiated Caco-2 cells prevents the production of the antimicrobial reagent, nitric oxide, in response to inflammatory cytokines (Maresca *et al*., 2005). Importantly, this inhibitory mechanism was related to a lack of activation of the NF-κB transcriptional factor that is crucial for inducing the expression of chemokines including IL-8, inflammatory cytokines, adhesion molecules and other antimicrobial reagents (Chen and Greene, 2004; Hayden and Ghosh, 2004).

Induction of IL-8 secretion in response to foreign antigens or cytokines requires ligand interaction with specific receptors, such as the Toll-like receptor (TLR)-5 for flagellin and tumour necrosis factor (TNF) receptor for TNFα, triggering the recruitment of scaffolding, adaptor and kinase proteins such as MyD88/IRAK/RIP and TRAF, to induce the phosphorylation-related activation of the IκB kinase (IKK) complex and mitogen-activated protein (MAP) kinases (Kobayashi *et al*., 2004; Meylan and Tschopp, 2005). IKK plays a crucial role in phosphorylating IκB, the cytoplasmic ‘inhibitory’ partner of NF-κB, triggering its ubiquitination and degradation to release NF-κB for translocation into the nucleus where its ability to transcribe specific genes is controlled by DNA and protein modifications (Chen and Greene, 2004; Hayden and Ghosh, 2004). An additional level of control occurs through the activation of MAP kinase members including the extracellular-regulated kinase (ERK) 1/2, p38 and Jun amino-terminal kinase (JNK) that regulate processes such as accessory transcriptional factor function and mRNA stability (Hoffmann *et al*., 2002; O'Neill, 2006).

In this paper we explore the idea, and provide evidence for EPEC possessing a mechanism to prevent infected epithelial cells from inducing the innate immune response cascade in a model system representing the natural site of infection. Insights are provided relating to the molecular mechanism by which EPEC mediates this inhibition from the bacterial and host perspectives. Importantly, we show that the NF-κB-dependent innate immune response pathway is effectively silenced before EPEC compromises epithelial barrier function thereby providing a plausible explanation for the absence of strong inflammatory responses in infected humans.

## Results

### Maximal IL-8 secretory response requires EPEC flagellin to access the basolateral surface

**Fig. 1:**
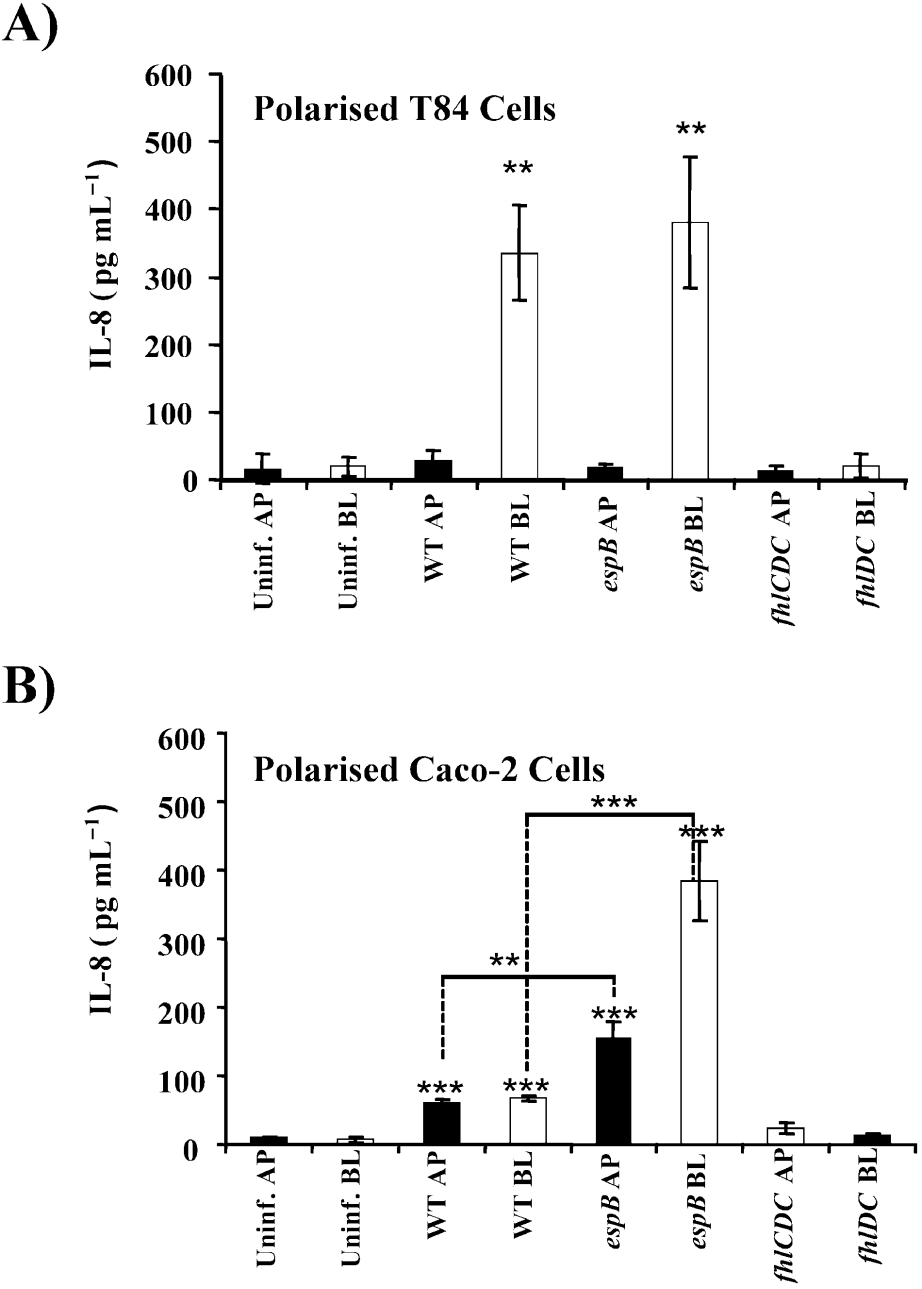
Maximal IL-8 secretory levels are dependent on access of EPEC flagellin to the basolateral surface of differentiated epithelia. The apical (AP; filled rectangles) or basolateral (BL; open rectangles) compartments of differentiated (A) T84 or (B) Caco-2 cells were untreated (Uninfect.) or inoculated for 6 h with pre-activated EPEC strains – wild type (WT), the *espB* effector delivery-defective mutant or *flhDC* flagella-deficient mutant – prior to measuring IL-8 levels in the basolateral compartment by sandwich ELISA as described in *Experimental procedures*. Data are from at least three independent experiments with error bars revealing standard deviation. Statistical significance was evaluated using Student's *t*-test comparing data points relative to corresponding uninfected controls. Additional comparison of linked WT and *espB* data sets, indicated by lines, revealed no statistical difference for T84-infected cells in contrast to Caco-2. *P < 0.05; **P < 0.01; ***P < 0.001.

Studies with non-differentiated cells have identified flagellin as the major EPEC antigen responsible for triggering NF-κB-dependent secretion of the inflammatory-related chemokine IL-8. However, such a response may not occur during apical infection of differentiated intestinal monolayers as the host's flagellin receptor, TLR-5, is considered to be excluded from this surface in model systems (Gewirtz *et al*., 2001). To investigate this, differentiated Caco-2 and T84 monolayers – models of small and large intestinal epithelia respectively (Pinto *et al*., 1983; Dharmsathaphorn and Pandol, 1986; Delie and Rubas, 1997; Kunzelmann and Mall, 2002) – were infected for 6 h with wild-type EPEC or mutants deficient in TTSS-dependent translocation (*espB*) or flagellin expression (*flhDC*) prior to enzyme-linked immunosorbent assay (ELISA) quantification of IL-8 levels in the basolateral compartment (see *Experimental procedures*). Interestingly, while apical infection of T84 cells failed to trigger significant increases in IL-8 release above basal levels in uninfected cells, introduction of the bacteria to the basolateral side induced a ∼30–50-fold increase (Fig. 1A). In contrast, infection at either Caco-2 surface induced IL-8 secretory responses though basolateral infection induced a more potent (∼2.5-fold greater) response than apical infection (Fig. 1B). Importantly, IL-8 secretion was not induced in either cell line with the *flhDC* mutant that delivers effector proteins (data not shown) but does not express flagella (Fig. 1A and B). Moreover, the significant reduction in IL-8 secreted levels following apical or basolateral infection of Caco-2 cells with the wild-type strain, compared with the effector delivery-defective (*espB*) mutant (Fig. 1B), is supportive of the existence of a specific inhibitory mechanism. Surprisingly, corresponding basolateral infections of T84 cells failed to reveal any significant inhibition of flagella-dependent IL-8 secretion (Fig. 1A), linked to a delay in the delivery and/or inhibitory activity of the responsible effector molecule(s). Thus, this datum not only identifies flagellin as the EPEC antigen responsible for triggering IL-8 secretion in Caco-2 and T84 intestinal models but clearly demonstrates a requirement for this antigen to access the basolateral surface to stimulate the most potent IL-8 secretory response. Moreover, the data are consistent with EPEC possessing an effector delivery-dependent mechanism that more rapidly inhibits flagellin-induced IL-8 secretion from the small, than large intestinal epithelia model systems.

### Enteropathogenic *E. coli* abolishes the capacity of differentiated Caco-2 cells to secrete IL-8 before epithelial barrier function is compromised

**Fig. 2:**
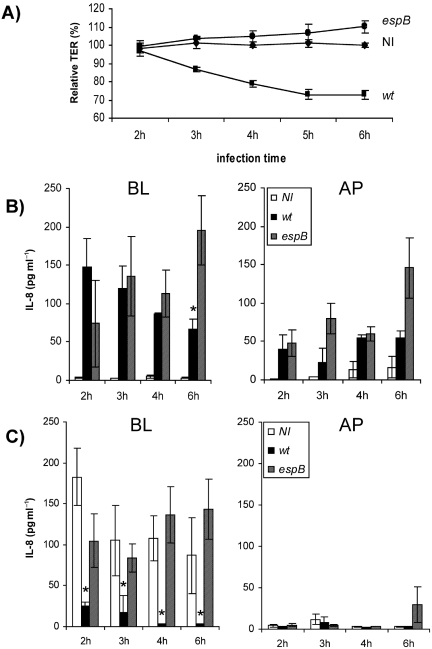
EPEC suppresses the ability of Caco-2 cells to secrete IL-8 before epithelial barrier function is compromised. The apical surface of differentiated Caco-2 was left uninfected (NI) or inoculated with pre-activate wild-type EPEC (WT) or the *espB* effector delivery-deficient mutant and (A) tight junction integrity was monitored at the indicated time points by measuring TER as described in *Experimental procedures.* In (B) the levels of IL-8 released into the basolateral (BL) or apical (AP) compartments at the indicated time points post inoculation were determined by sandwich ELISA as described in *Experimental procedures*. In (C) monolayers were treated for 1 h with bactericidal levels of gentamicin (100 μg ml^−1^ final concentration) to end the infection at the indicated time points. The following day (∼16 h later) TNFα (10 ng ml^−1^ final concentration) was added for 2 h to the basolateral compartment and the levels of IL-8 released was measured by sandwich ELISA. Data are from at least three independent experiments with error bars revealing standard deviation. Statistical significance was evaluated using Student's *t*-test comparing data points relative to (B) corresponding 2 h infection data point or (C) to corresponding uninfected control. *P < 0.05; **P < 0.01; ***P < 0.001.

Given the absence of a strong inflammatory response in EPEC-infected humans, despite the pathogen's ability to disrupt barrier function, we examined the prediction that loss of barrier integrity does not lead to increases in flagellin-mediated IL-8 secretion. As previously published (Dean and Kenny, 2004), a 6 h infection with the effector delivery-defective mutant (*espB*) did not disrupt Caco-2 barrier function, in contrast to the wild-type strain where a significant loss was first evident 3 h post infection with progressively greater defects over the 6 h infection period (Fig. 2A). Consistent with earlier results (Fig. 1B), very low levels of IL-8 were detected in the basolateral well of uninfected cells (Fig. 2B), with increased IL-8 levels following 6 h apical infections. Interestingly, apical infection also triggered IL-8 release into the apical well, though the levels are approximately one-third of those within the basolateral well (Fig. 2B). Again consistent with previous data (Fig. 1B), IL-8 levels were reduced in cells infected with the wild-type strain compared with the *espB* mutant (Fig. 2B). Infection for 2, 3, 4 and 6 h with the *espB* mutant was associated with no, or small, changes in the asymmetric distribution of IL-8 levels between both apical and basolateral compartments. In contrast, there was a gradual loss in the asymmetric distribution of IL-8 (observed 2 h post infection) over the following 4 h infection period with the wild-type strain (Fig. 2B). This loss in asymmetric distribution parallels disruption of barrier function (Fig. 2A) indicating that the latter process, i.e. barrier dysfunction enables macromolecules, such as IL-8, to diffuse across the barrier. The clear absence of increased IL-8 secretion in response to EPEC-mediated barrier dysfunction, which also allows access of host membrane proteins to the apical surface (Muza-Moons *et al*., 2003), is suggestive of a defect in inducing IL-8 secretion downstream of TLR-5/flagellin interaction.

To explore this premise we tested whether the cytokine TNFα can induce IL-8 secretion from Caco-2 cells pre-infected with wild-type EPEC or the *espB* effector delivery-defective strain. Figure 2C shows that uninfected cells and cells pre-infected with the *espB* mutant for 2, 3, 4 or 6 h secreted similar levels of IL-8 in response to the basolateral addition of TNFα (Fig. 2C). In contrast, cells pre-infected with wild-type EPEC for 3, 4 or 6 h, when barrier function was detectably compromised (Fig. 2A), failed to release significant amounts of IL-8 (Fig. 2C). Moreover, significantly reduced levels of IL-8 was secreted from cells pre-infected with wild-type EPEC for 2 h (∼25 pg ml^−1^ compared with 100–180 pg ml^−1^ for uninfected and *espB* pre-infected cells; Fig. 2C), when barrier dysfunction is not evident (Fig. 2A), clearly demonstrating that EPEC possesses an effector delivery-dependent mechanism that inhibits IL-8 secretion from differentiated Caco-2 before barrier function is compromised.

### Role for non-LEE-encoded effectors in inhibiting IL-8 secretion

**Fig. 3:**
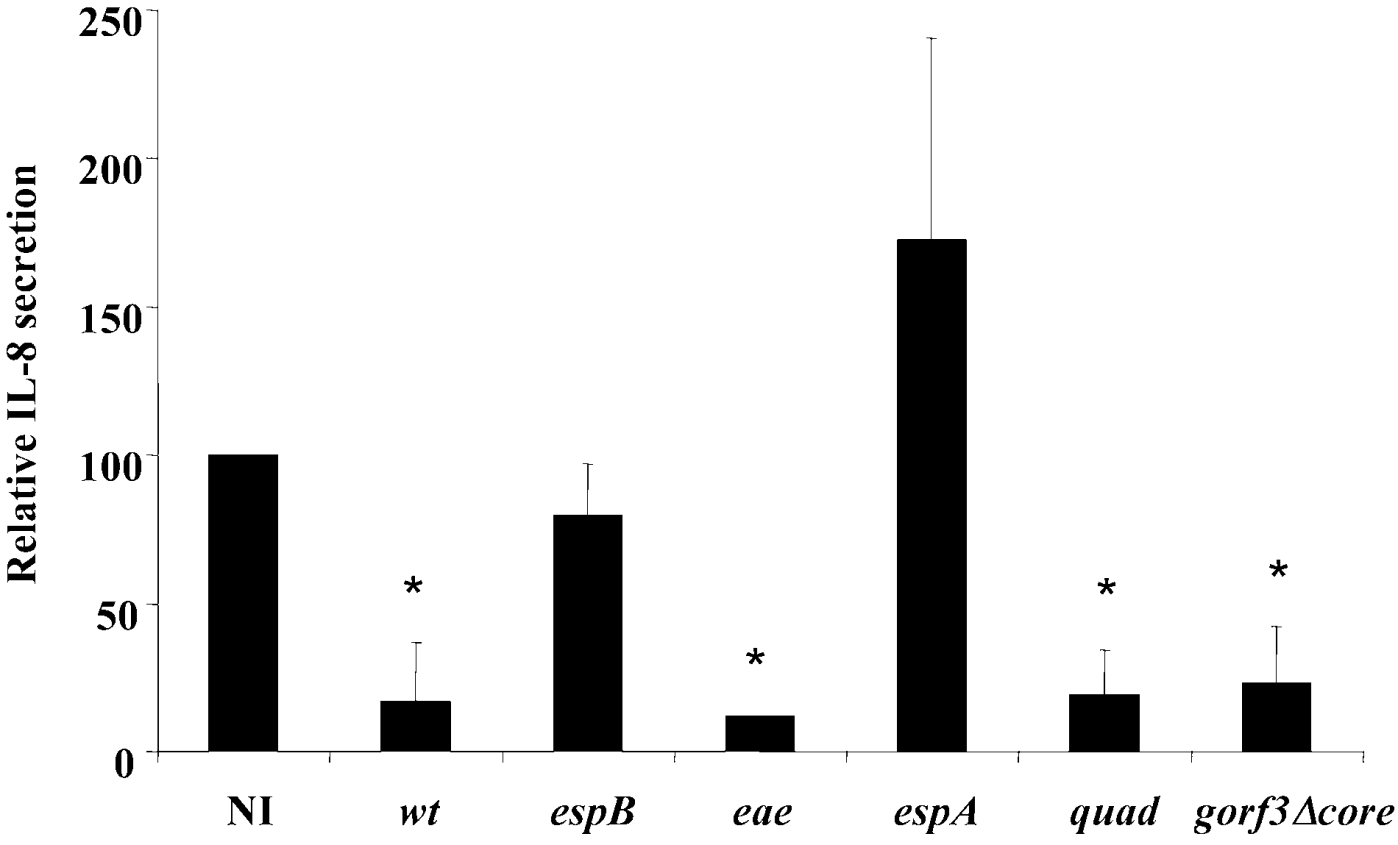
Role of LEE-encoded effectors in inhibiting TNF-induced IL-8 secretion. The apical surface of differentiated Caco-2 cells was left uninfected (NI) or inoculated for 3 h with pre-activated EPEC – wild type (WT), *espB* or *espA* (effector delivery-deficient), *eae* (Intimin-deficient), *quad* (Map, EspF, Tir and Intimin-deficient) or *gorf3*Δcore (deletion of LEE region carrying *espH*, *cesF*, *map*, *cesT*, *tir* and *eae* genes in an *espGorf3* double mutant background) strains. Infected monolayers were treated for 1 h with bactericidal levels of gentamicin (100 μg ml^−1^ final concentration) and the following day (∼16 h later) TNFα (10 ng ml^−1^ final concentration) was added to the basolateral compartment for 3 h prior to measuring the levels of IL-8 released into this compartment by sandwich ELISA. Data are from at least three independent experiments with error bars revealing standard deviation. Statistical significance evaluated using Student's *t*-test comparing data to uninfected cells. *P < 0.05.

Given the TTSS effector delivery-dependent nature of the inhibition, we screened available mutants to identify genes involved in the inhibitory process. These analyses confirmed the essential role of genes required for effector delivery, including *espA* and *espB* (Fig. 3), while dismissing crucial roles for the LEE-encoded EspF, EspG, EspH, Map, Tir, Intimin and non-LEE-encoded EspG homologue, Orf3 (Fig. 3, and data not shown). Given the redundant nature of Map, EspF, Tir and Intimin in subverting host cellular processes (Dean *et al*., 2005; 2006) we investigated, but failed to identify cooperative roles using a quadruple (*quad*) mutant missing all four gene products (Fig. 3). Cooperative roles for all known LEE-encoded effectors and four non-LEE effectors were assessed with a *gorf*3Δcore strain in which a central ‘core’ region was deleted from the LEE operon in an *espGorf3* double mutant background (see *Experimental procedures*) producing a strain not expressing EspG, Orf3, EspH, CesF, Map, Tir, CesT or Intimin. CesT is a chaperone required for the efficient translocation and/or effector function of Map and Tir (Abe *et al*., 1999; Creasey *et al*., 2003) and more recently reported to be a chaperone for EspZ, NleA, NleF and NleH (Thomas *et al*., 2005) while CesF is the EspF chaperone (Elliott *et al*., 2002). Thus, while the *gorf*3Δcore mutant no longer encodes EspG, Orf3, EspH, Map, Tir or Intimin, the additional absence of the CesT/CesF chaperones is predicted to inhibit the translocation and/or effector function of the EspF, EspZ, NleA, NleF and NleH proteins. As this strain was as effective as wild-type EPEC at inhibiting IL-8 secretion (Fig. 3) this suggests that other non-LEE-encoded effector(s) are responsible for the inhibitory effect. Moreover, inhibition of IL-8 secretion by mutants unable to disrupt barrier function (e.g. *eae*-negative mutant) reveals that both processes are not linked.

### Enteropathogenic *E. coli* prevents NF-κB transcriptional factor activation and translocation into the nucleus

**Fig. 4:**
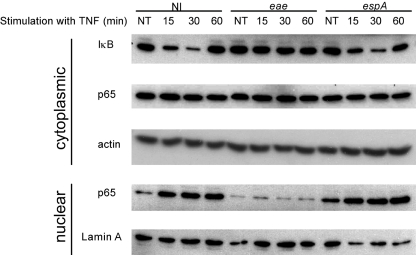
EPEC prevents the activation and translocation of a transcriptional factor crucial to IL-8 gene transcription. The apical surface of differentiated Caco-2 was left uninfected (NI) or inoculated for 3 h with pre-activated *eae* (Intimin-deficient) or *espA* (effector delivery-defective) mutants prior to ceasing infections by treating for 1 h with bactericidal levels of gentamicin (100 μg ml^−1^ final concentration). TNFα (10 ng ml^−1^ final concentration) was then added to the basolateral compartment for 0 (non-treated; NT), 15, 30 and 60 min before isolating cytoplasmic and nuclear extracts as described in *Experimental procedures*. Equal amounts of protein extract were resolved by SDS-PAGE (12%), transferred to nitrocellulose and probed with anti-IκB or anti-p65 antibodies as well as anti-actin and anti-Lamin A as loading controls for cytoplasmic and nuclear proteins respectively. Provided blot is representative of those obtained from three independent experiments.

TNFα-mediated IL-8 secretion is dependent on a phosphorylation signalling cascade leading to the degradation of the IκB inhibitory protein to release NF-κB for translocation into the nucleus (Hoffmann *et al*., 2002; O'Neill, 2006). To investigate whether the IκB degradation or NF-κB translocation processes are altered by EPEC, differentiated Caco-2 cells were apically infected for 3 h with the Intimin-negative *eae* strain that inhibits IL-8 secretion (Fig. 3), without compromising barrier function (Dean and Kenny, 2004), or the *espA* (effector delivery-defective) mutant. The bacteria were killed (see *Experimental procedures*) and cells basolaterally treated with TNFα for 0, 15, 30 and 60 min before isolating ‘cytoplasmic’ and ‘nuclear’ fractions for Western blot analysis. Figure 4 reveals the rapid, but transient degradation of IκB and subsequent accumulation of NF-κB (p65 isoform) in the nuclear fraction of uninfected and *espA* pre-infected cells. In contrast, cells pre-infected with the *eae* mutant showed no such response, displaying patterns indistinguishable from uninfected untreated cells (Fig. 4). Thus, EPEC inhibition of IL-8 secretion is linked to the absence of NF-κB translocation into the nucleus presumably related to the observed defect in IκB degradation.

### Enteropathogenic *E. coli* inhibits IKK-mediated phosphorylation of IκB and NF-κB

**Fig. 5:**
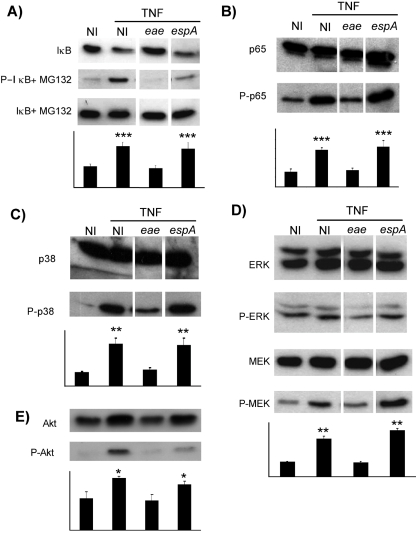
EPEC inhibits phosphorylation-associated activation of IKK, ERK, p38 and PI3-K pathways. Differentiated Caco-2 cells were (B–E) left untreated or (A) pretreated, or not, with the proteasome inhibitor MG132 (50 μM final concentration) for 1 h prior to 3 h apical inoculations with pre-activated *eae* (Intimin-deficient) or *espA* (effector delivery-defective) mutants. Infections were stopped by 1 h treatment with bactericidal levels of gentamicin (100 μg ml^−1^ final concentration) prior to adding TNFα (10 ng ml^−1^ final concentration) for 30 min to the basolateral compartment of uninfected (NI) or infected cells followed by the isolation of Triton X-100 (1% v/v) soluble extracts as described in *Experimental procedures*. Equal protein loadings were resolved by SDS-PAGE (12%), transferred to nitrocellulose and probed with antibodies specific to (A) IκB or its phosphorylation on serine 32, P-IκB, (B) p65 isoform of NF-κB or its phosphorylation on serine 536, P-p65, (C) p38 Map kinase or its phosphorylation on threonine180/tyrosine182, P-p38, (D) ERK1/2 MAP kinase or phosphorylation on threonine202/tyrosine204 (P-Erk) and MEK MAP kinase kinase or phosphorylation on serine 221 (P-MEK) respectively, and (E) Akt or its phosphorylation on serine 473 (P-Akt). Linked graphs show relative amplitude of phosphorylated bands (arbitrary units) compared with those of the corresponding uninfected untreated cells obtained by densitometry examination of data from three independent experiments with standard deviation indicated by error bars. Statistical significance evaluated using Student's *t*-test comparing data with that of the corresponding uninfected untreated cells. *P < 0.05; **P < 0.01; ***P < 0.001.

IκB ubiquitination-dependent degradation requires phosphorylation by the IKK complex (Hoffmann *et al*., 2002). To assess the effect of EPEC infection on this process, IκB phosphorylation was induced with TNFα, in the presence or absence of the proteasomal inhibitor, MG132, in cells pre-infected for 3 h with EPEC (*eae* or *espA* mutants). Western analysis of cellular extracts for phosphorylated (on serine 32) or total IκB revealed rapid phosphorylation- (Fig. 5A middle panel and graph) and proteasomal-dependent degradation (Fig. 5A top versus bottom panels) in uninfected and *espA*-infected cells. In contrast, pre-infection with the *eae* mutant prevented IκB phosphorylation and thus degradation in response to TNFα treatment (Fig. 5A).

To further assess the functionality of the IKK complex we probed for the phosphorylation of NF-κB (p65 isoform) on serine 536, as this modification is linked to its activation (Chen and Greene, 2004). Similar results were obtained to those with IκB (Fig. 5A), as TNFα treatment of uninfected or *espA*-infected cells triggered the phosphorylation of NF-κB, unlike cells pre-infected with the *eae* mutant (Fig. 5B). Thus, the ability of effector translocation-competent EPEC strains to prevent IL-8 secretion is linked to a defect in IKK-mediated phosphorylation of IκB and NF-κB thereby preventing both the activation and nuclear translocation of NF-κB for gene transcription.

### Enteropathogenic *E. coli* inhibits multiple MAP kinase pathways

IL-8 secretion is also regulated at *trans*-activational and mRNA stability levels through phosphorylation-related activation of MAP kinase family members including ERK1/2, JNK and p38 kinases (Chen and Greene, 2004; Hayden and Ghosh, 2004). Assessment of the activation-related phosphorylation of ERK1/2 and p38 Map kinases, as well as the upstream MEK kinase, produced similar results to those observed with IκB and NF-κB (Fig. 5). Thus, TNFα treatment of uninfected and *espA*-infected cells induced the phosphorylation of all three kinases unlike cells pre-infected with the *eae* mutant where no corresponding phosphorylation was detected (Fig. 5C and D). The phosphorylation status of JNK was not assessed as examination of total cellular levels revealed its unexpected degradation in response to TNFα treatment in cells pre-infected with the *eae* mutant unlike uninfected cells or those pre-infected with the *espA* mutant (data not shown).

As EPEC inhibit the phosphatidylinositol 3-kinase (PI3-K)/Akt pathway in phagocytic cell lines (Celli *et al*., 2001; Quitard *et al*., 2006), with this pathway implicated in TNFα-mediated NF-κB activation (Reddy *et al*., 2000), we assessed its activity in TNFα-treated differentiated Caco-2 by probing for the activation-associated phosphorylation of Akt on serine 473. Figure 5E illustrates TNFα-dependent Akt phosphorylation in uninfected cells and cells pre-infected with the *espA* (effector delivery-defective) mutant. In contrast, cells pre-infected with the *eae* mutant, which inhibits NF-κB-dependent IL-8 secretion (Fig. 3), fail to induce Akt phosphorylation in response to TNFα treatment. Thus, EPEC delivery of unidentified effector proteins into differentiated Caco-2 cells inhibit the phosphorylation-related activation of key kinases of the IKK, Map kinase and PI3-K pathways that play crucial roles in NF-κB-dependent transcription.

## Discussion

It has been, and continues to be argued that EPEC interaction with epithelia cells leads to a pro-inflammatory response (Savkovic *et al*., 1996; 1997; 2001; 2003; Czerucka *et al*., 2001; Zhou *et al*., 2003; Borthakur *et al*., 2006), though the extent of this may depend on a balance between pro- and anti-inflammatory signals (Hauf and Chakraborty, 2003; Chen and Frankel, 2005; Sharma *et al*., 2006). However, this interpretation is based on studies using non-differentiated epithelia cells where the TLR-5 receptor that detects flagellin, the major EPEC pro-inflammatory antigen, is not restricted from the site of host–bacterial interaction unlike differentiated T84 epithelia (Gewirtz *et al*., 2001). In support of this premise T84 cells (model of large intestinal epithelia) fail to secrete IL-8 in response to a 6 h apical EPEC infection while basolateral infection triggers a potent IL-8 secretory response (Fig. 1A). Thus, induction of an IL-8 pro-inflammatory secretory response is dependent on EPEC antigens gaining access to the basolateral membrane in this model system. In contrast, apical infection of differentiated Caco-2 cells (model of small intestinal epithelia) induced IL-8 secretion but to a lesser extent (∼2.5-fold less) than corresponding basolateral infections (Fig. 1B). This datum is consistent with *in vivo* data (Cario and Podolsky, 2000; Bambou *et al*., 2004) indicating the presence of TLR-5 on the apical membrane of small intestinal epithelia and supported by the finding that these IL-8 secretory responses were dependent on EPEC-expressing flagella (Fig. 1). Our finding contrasts with the report that addition of purified flagellin (from the closely related pathogen, EHEC) to apical or basolateral surfaces induced similar levels of IL-8 secretion (Miyamoto *et al*., 2006). This discrepancy presumably reflects differences in antigen origin, concentration and/or the presentational context of purified versus bacterial-associated flagellin. Moreover, our models fail to provide supportive evidence for other non-flagellin antigens in stimulating IL-8 secretion (Sharma *et al*., 2006), possibly reflecting differences in cell types (Caco-2/T84 in this study versus HT-29) or an apparent non-differentiated status of the HT-29 cells (Zweibaum *et al*., 1985). However, our studies clearly identify flagellin as the EPEC antigen triggering IL-8 secretion from differentiated Caco-2 and T84 cells, with basolateral infections triggering more potent IL-8 secretory responses than apical infections.

The finding that significantly less IL-8 was released following apical or basolateral infection of Caco-2 cells with the wild-type strain compared with the effector delivery-defective mutant (Fig. 1B) supported the existence of an effector-dependent mechanism to antagonize flagellin-induced IL-8 secretion (Sharma *et al*., 2006). This premise was further supported by the fact that EPEC TTSS-dependent disruption of barrier function (Fig. 2A), which facilitates the movement of ions, macromolecules (including IL-8; Fig. 2B) and membrane proteins to the apical surface (Muza-Moons *et al*., 2003), was not linked to increases in IL-8 secretion levels (Fig. 2B). Moreover, cells whose barrier function had been compromised by EPEC failed to secrete IL-8 in response to the basolateral addition of TNFα, unlike uninfected cells or those infected with an effector delivery-defective mutant (Fig. 2B and C). Informatively, cells pre-infected with the wild-type strain for 2 h, before barrier dysfunction was evident (Fig. 2A), released four to five times less IL-8 in response to TNFα than uninfected cells or those pre-infected with the effector delivery-defective mutant (Fig. 2B). Crucially, cells pre-infected with the *eae* (Intimin-deficient) mutant, which does not disrupt barrier function (Dean and Kenny, 2004), failed to secrete IL-8 in response to the basolateral addition of TNFα (Fig. 3), flagellin and other undefined non-flagellar EPEC antigens (antigens released following gentamicin killing of the *flhDC* mutant that induce potent IL-8 secretory responses in uninfected cells; data not shown). The viability of these pre-infected cells was evidenced by retention of tight junction integrity 24 h after ceasing bacterial infection as well as the ability of such cells to phosphorylate the STAT-1 transcriptional factor in response to cytokines (Maresca *et al*., 2005). It should be stressed that EPEC-mediated barrier dysfunction and inhibition of IL-8 secretion are unlinked, as the former (Dean and Kenny, 2004) but not the latter is dependent on LEE-encoded Intimin protein (Fig. 3). Thus, this datum reveals that EPEC interaction with the apical surface of differentiated Caco-2 cells inactivates the capacity of host Toll-like and TNF receptors to induce IL-8 secretory responses before other EPEC effector-mediated signalling has compromised barrier function.

The above finding has important implications in relation to the infection process as it provides a plausible explanation to why EPEC infection of humans is only associated with rather weak inflammatory responses (Ashkenazi *et al*., 1983; Miller *et al*., 1994; Nataro and Kaper, 1998) despite the pathogen's *in vitro* and *in vivo* defined capacity to disrupt epithelial barrier function (Dean and Kenny, 2004; Shifflett *et al*., 2005; Ma *et al*., 2006). However, this raises a question on why closely related members including rabbit-EPEC and mouse-specific *Citrobacter rodentium* are associated with inflammatory diseases. This may be due to differences in host physiology, infection site or the number, amino acid composition and strain-specific nature of some TTSS-dependent effectors (Perna *et al*., 1998; Deng *et al*., 2004; Tobe *et al*., 2006). Thus, it is likely that strains have acquired and/or adapted specific effectors, or encoding functions, to mediate distinct strategies that maximize their survival, replication and dissemination potential in their host.

While our studies clearly demonstrated the TTSS delivery-dependent nature of the inhibition of IL-8 secretion, we failed to identify the responsible effector(s). However, we ruled out important roles, individual or cooperative, for the LEE-encoded Map, EspF, Tir effectors as well as the Intimin membrane protein (Fig. 3) that function together in various combinations to trigger disease-related changes in cell physiology (Dean and Kenny, 2004; Dean *et al*., 2006). Moreover, roles were not detected for LEE-encoded EspG and EspH proteins, the non-LEE EspG homologue Orf3 or the non-LEE non-TTSS-secreted EspC enterotoxin (Navarro-Garcia *et al*., 2004) (Fig. 3; data not shown). In addition, our studies are consistent with no significant role for 11 effectors (all known LEE-encoded effectors, Intimin and the non-LEE-encoded Orf3, NleA, NleF and NleH effectors) in the inhibitory process. However, this premise is based on the rationale that mutants missing the CesT and CesF chaperones required for the efficient translocation of Map/Tir and EspF into host cells, respectively, fail to induce cellular changes linked to these effectors (Abe *et al*., 1999; Elliott *et al*., 2002; Creasey *et al*., 2003) and thus the supposition that EspZ, NleA, NleF and NleH would have similar defects given that CesT is reported to be their chaperone (Thomas *et al*., 2005). Thus, deleting the LEE region encompassing *espH*, *cesF*, *map*, *tir*, *cesT* and *eae* genes from an *espG*/*orf3* double mutant background removes the genes encoding EspG, Orf3, EspH, Map, Tir and Intimin, with the additional absence of genes encoding CesT/CesF inhibiting EspF, EspZ, NleA, NleF and NleH effector translocation and/or function. Thus, our results suggest that the inhibitory effect is likely to be mediated by one or more of an increasing list of non-LEE TTSS-dependent effectors (Spears *et al*., 2006; Tobe *et al*., 2006).

We also provided important new insights into the inhibitory mechanism within a model representing the natural infection site of EPEC and which, consistent with other studies (Hauf and Chakraborty, 2003; Maresca *et al*., 2005), includes a central role for the NF-κB transcription factor that plays a crucial role in inducing innate immune responses (Chen and Greene, 2004; Hayden and Ghosh, 2004). However, our investigations have unveiled a more complicated situation whereby EPEC inhibits multiple phospho-relay cascades involved in the activation of kinases of the IKK, MAP kinase (ERK1/2, p38 and JNK) and PI3-K pathways (Figs 4 and 5) linked to NF-κB nuclear translocation, transcriptional activation, mRNA stability, protein expression and delivery (Hoffmann *et al*., 2002; Chen and Greene, 2004; Hayden and Ghosh, 2004; O'Neill, 2006). Thus, while we demonstrate that pre-infection of host cells with an effector delivery-defective mutant had no significant impact on the ability of TNFα to activate these pathways, cells pre-infected for 3 h with effector delivery-competent strains were effectively unresponsive to this cytokine. Moreover, the inhibitory mechanism was linked to a lack of phosphorylation-associated activation of IKK, MAP kinase (ERK1/2, p38), PI3-K, NF-κB and IκB (Figs 4 and 5). Unexpectedly the MAP kinase, JNK, was found to be degraded in a proteasomal-independent manner in cells pre-infected with the *eae* mutant prior to TNFα treatment, unlike uninfected cells or those pre-infected with an effector delivery-defective strain (data not shown). The non-responsiveness of these signalling pathways would explain why various bacterial antigens (released from gentamicin-killed EPEC) and cytokines (TNFα, IL-1β, IFNγ) fail to induce NF-κB-dependent IL-8 secretion (Fig. 2) or NO production (Maresca *et al*., 2005). Thus, it would appear that EPEC rapidly desensitizes infected cells from responding to external signals that would normally trigger NF-κB-dependent innate immune responses. As suggested above, this might explain why EPEC infection of humans is not associated with strong inflammatory responses linked with breaches in epithelial barrier integrity (Mullin *et al*., 2005). Inhibiting NF-κB-dependent function not only prevents the induction of IL-8 (this study) and NO production (Maresca *et al*., 2005) but should also inhibit the induced expression of other chemokines, cytokines, adhesion molecules, ion transporters and antimicrobial enzyme activities (Chen and Greene, 2004; Hayden and Ghosh, 2004) thereby hindering the host's capacity to rapidly counter EPEC infection.

Given the essential nature of NF-κB activity in the innate immune response it is not surprising that many pathogens have evolved mechanisms that partially or completely alter its activity. Indeed, pathogenic factors can interfere at specific or multiple steps of one or more of the above described pathways by providing factors often structurally and/or functionally analogous to host regulatory proteins (Tato and Hunter, 2002). It is evident from our studies that EPEC inactivates several pathways that regulate NF-κB function akin to the *Yersinia* YopJ TTSS-injected effector that transacetylates residues linked to phosphorylation-related activation of MAP kinase kinase members (Mukherjee *et al*., 2006). However, it is unlikely that EPEC utilizes a similar mechanism as YopJ family members have a conserved catalytic domain with no homologues reported for EPEC or EHEC (Perna *et al*., 2001; Tobe *et al*., 2006). Intriguingly, EHEC encodes several effectors (NleH) that share homology to the *Shigella* OspG TTSS-injected effector protein that interferes with NF-κB signalling (Kim *et al*., 2005). However, these are unlikely to represent the EPEC inhibitory factor(s) as (i) the *espGorf3*Δcore mutant inhibits IL-8 secretion although it should be defective in NleH translocation (see above, note only one of two predicted EPEC NleH genes appears intact) and (ii) OspG function to delay, not prevent IκB degradation. Studies are currently underway to define the effector molecule(s) responsible for inhibiting the NF-κB-related pathways and the molecular mechanism of inhibition.

In summary, our studies with the extensively used Caco-2 model of small intestinal epithelia clearly demonstrates that apical infection with EPEC triggers a rapid TTSS-dependent inhibition of signalling pathways crucial for the induction of the innate immune response responsible for producing chemokines, cytokines and antimicrobial activities. This work not only argues against a role for 11 known effector proteins but also unveils a complex inhibitory mechanism encompassing the IKK, MAP (ERK1/2, p38, JNK) kinase and PI3-K signalling pathways. Importantly, the inactivation of these pathways precedes EPEC-induced barrier disruption providing a plausible explanation for the weak inflammation response in infected humans.

## Experimental procedures

### Bacterial strains, growth condition and cell culture

Bacterial strains used in this study are listed in Table 1. All strains were grown from single colonies on Luria–Bertani (LB) plates in LB broth plus or minus nalidixic acid (25 μg ml^−1^ final) at 37°C in a 5% CO_2_ atmosphere overnight without shaking. Caco-2 cells (ATCC No HTB-37) and T84 cells (ATCC No CCL-248) were grown at 37°C with 5% CO_2_ in Dulbecco's minimal Eagle's medium (DMEM) supplemented with 10% heat-inactivated fetal calf serum, 1% nonessential amino acids, 2 mM l-glutamine and 1% antibiotics (penicillin/streptomycin, 100 units ml^−1^ final concentration; Sigma). Cells were used between passages 10 and 40 and seeded onto 1 (0.45 μm pores) or 4.5 (3 μm pores) cm^2^ Transwell-Clear inserts (Corning) at a density of 250 000 cells cm^−2^. Cells were differentiated over a 7 day period with differentiation, which depends on tight junction formation, routinely verified using an EVOM voltohmeter (World Precision Instruments) to measure transepithelial electrical resistance (TER) across the junctions before and after infection.

**Table 1:** EPEC strains and oligonucleotides used in this study.

Strain	Deficiency	Reference
E2348/69; O127:H6 serotype	None	Levine *et al*., (1985)
*sep*-2 (CVD452)	TTSS	Jarvis *et al*., (1995)
*eae* mutant (CVD206)	Intimin	Donnenberg and Kaper (1991)
*espA* mutant	EspA	Kenny *et al*. (1996)
*espB* mutant (UMD864)	EspB	Donnenberg *et al*. (1993)
*espC* mutant	EspC	Stein *et al*. (1996)
*espF* mutant	EspF	Warawa *et al*. (1999)
*espG*/*orf3* mutant	EspG/Orf3	Elliott *et al*. (2001)
*espH* mutant	EspH	Tu *et al*. (2003)
*map* mutant	Map	Kenny and Jepson (2000)
*tir* mutant	Tir	Kenny *et al*. (1997)
*quad* mutant	Tir/Intimin/EspF/Map	Quitard *et al*. (2006)
*flhDC* mutant (DF971)	*Flagella*	Ilan Rosenshine
*espG/orf3Δcore*	EspG/Orf3/EspH/CesF/Map/CesT/Tir/Intimin	This study

Oligonucleotide	Sequence (5′ to 3′ with incorporated restriction sites underlined or in italics)
UpcorePS	GTTGTTACATCGGGAGGATTTGCGTGCGGGCGGCA
UpcoreNS	CGGAATTC*AGATCT*GATTAATCACATACTACGCTAAAGT
DowncorePS	AGGAATTC*AGATCT*AATGCTTATGCCACTTGTGTAAAATAAA
DowncoreNS2	GCAGCTCGAGCATTTCATTGAGCACCTTCTCGA

Note all mutants except *espC*, *espH* are nalidixic resistant with a nalidixic resistant isolate of the *flhDC* mutant selected for these studies.

### Generation of *espG/orf3Δcore* mutant

An ∼2 kb region immediately upstream of *espH* was PCR amplify using the high-fidelity Pfu polymerase (Promega) and an oligonucleotide pair (Table 1) incorporating BglII/EcoRI sites at the 3′ end. Similarly, an ∼2 kb region immediately downstream of the *eae* (Intimin) gene was amplified with an oligonucleotide pair (Table 1) incorporating BglII/EcoRI sites at the 5′ end and an XhoI site at the 3′ end. The A-addition kit (Qiagen) was used to add A-tails to the PCR fragments prior to ligating into pDrive (Qiagen). The upstream region was cloned on an XbaI (upstream of 5′ end within cloning vector)/EcoRI fragment together with the downstream EcoRI/XhoI fragment into the XbaI/SalI (SalI compatible with XhoI) sites of the pKNG101 suicide vector (Kaniga *et al*., 1991). The plasmid was isolated from *E. coli* SY327 and transformed into strain SM10 prior to conjugating with the EPEC *espG*/*orf3* mutant, as previously described (Kenny *et al*., 1997), to introduce the streptomycin-resistant (50 μg ml^−1^ final concentration) suicide plasmid. Streptomycin-resistant clones having undergone allelic exchange leading to the replacement of the wild-type locus with that missing the *espH*, *cesF*, *map*, *cesT*, *tir* and *eae* genes were selected on LB plates without NaCl containing 5% sucrose, as previously described (Kaniga *et al*., 1991). SDS-PAGE Coomassie and Western blot analyses confirmed that the *espG/orf3*Δ*core* mutant clone expressed and secreted wild-type levels of the EspA,B,D translocator proteins in the absence of Map, Tir or Intimin with EspF secreted levels reduced due to the absence of its chaperone, CesF (data not shown).

### Infection protocol

At least 2 h before infection the medium in the apical and basolateral compartments was replaced with DMEM (without supplements). Overnight bacterial cultures were diluted (1:10) in non-supplemented DMEM and left at 37°C in 5% CO_2_ atmosphere for 3 h to pre-activate the bacteria (Dean *et al*., 2006), by which time the OD_600_ typically reached 0.2–0.3. An appropriate volume for a final infection of 50 bacteria per host cell was added to the apical or basolateral compartment, as indicated, for various times. For basolateral infections (3 μm pore inserts), bacteria were added for 1 h (total volume 100 μl) to the porous surface of inverted inserts before re-inverting into fresh DMEM for continued incubation. In some experiments, infection was stopped by the addition of gentamicin (100 μg ml^−1^ final concentration) for 1 h before the basolateral addition of TNFα (10 ng ml^−1^ final concentration), bacterial supernatants or gentamicin-killed EPEC.

### Measurement of IL-8 secretion levels

The level of IL-8 released into the apical and/or basolateral compartments was assessed using an ELISA kit (DB Biosciences) following the manufacture's directions.

### Isolation of soluble, cytoplasmic and nuclear extracts

Following bacterial infection and/or stimulation, inserts were washed with cold PBS and cells lysed in 1% Triton X-100/PBS buffer containing a cocktail of protease inhibitors (Sigma; P8340 1:1000 dilution), 1.25 mM sodium fluoride (NaF), 1 mM sodium orthovanadate (NaVO_4_) and 1 mM phenylmethylsulphonylfluoride (PMSF). Centrifugation (13 000 *g*, 4°C, 5 min) pelleted bacteria, host nuclei and cytoskeleton with the remaining ‘soluble’ fraction resuspended in Laemmli sample buffer (Laemmli, 1970). In other experiments washed cells were scraped into cold PBS before resuspending in a buffer containing 10 mM Hepes pH 7.8, 10 mM KCl, 2 mM MgCl_2_, 0.1 mM EDTA, 1 mM DTT, 1.25 mM NaF, 1 mM NaVO_4_, 1 mM PMSF for 15 min on ice. Nonidet P-40 (0.5% final concentration) was added and the mixture vortexed for 15 s prior to centrifugation (13 000 *g*, 4°C, 30 s) to pellet the host nuclei. The soluble supernatants samples were resuspended in Laemmli sample buffer. The pelleted nuclei were resuspended in a buffer containing 50 mM Hepes pH 7.8, 50 mM KCl, 300 mM NaCl, 10% glycerol, 0.1 mM EDTA, 1 mM DTT, 0.2 mM NaF, 0.2 mM NaVO_4_, 1 mM PMSF and agitated for 20 min at 4°C, vortexing briefly every 5 min. The samples were centrifuged (13 000 *g*, 4°C, 5 min) and supernatants containing nuclear proteins resuspended in Laemmli sample buffer. Samples were boiled for 5 min and analysed immediately or stored at −80°C.

### Immunoblotting

Equal volumes of cellular samples were resolved by SDS-PAGE (12%), transferred to nitrocellulose and blocked for 1 h in 5% ‘Blotto’ skimmed milk powder in PBS. Blots were incubated overnight at 4°C with gentle rocking in a 5% bovine serum albumin/PBS solution containing antibodies (from Santa Cruz) against IκBα, p38, NF-κB (p65 isoform) or (from Cell Signalling) phospho-NF-κB (p65 isoform; Ser 536), phospho-IκBα (Ser 32), phospho-p38 (Thr180/Tyr182), ERK, phospho-ERK (Thr202/Tyr204), MEK and phospho-MEK (Ser221). Extensively washed blots were incubated with the appropriate horseradish peroxidase-conjugated secondary antibody and developed in Super Signal West Pico chemiluminescent substrate (Pierce) with the signal detected on Hyperfilm ECL (Amersham Biosciences) following manufactures' recommendations.
